# Interdisciplinarity in medical education research: myth and reality

**DOI:** 10.1007/s10459-020-09977-8

**Published:** 2020-06-24

**Authors:** Mathieu Albert, Paula Rowland, Farah Friesen, Suzanne Laberge

**Affiliations:** 1 Wilson Centre, Department of Psychiatry, University of Toronto, University Health Network, Toronto, Canada; 2 Wilson Centre, Department of Occupational Science and Occupational Therapy, University of Toronto, University Health Network, Toronto, Canada; 3 Centre for Faculty Development, Faculty of Medicine, University of Toronto at St. Michael’s Hospital, Unity Health Toronto, Toronto, Canada; 4 School of kinesiology and physical activity sciences, Université de Montréal, Montreal, Canada

**Keywords:** Interdisciplinarity, Disciplines, Medical education research, Citation analysis

## Abstract

The medical education (Med Ed) research community characterises itself as drawing on the insights, methods, and knowledge from multiple disciplines and research domains (e.g. Sociology, Anthropology, Education, Humanities, Psychology). This common view of Med Ed research is echoed and reinforced by the narrative used by leading Med Ed departments and research centres to describe their activities as “interdisciplinary.” Bibliometrics offers an effective method of investigating scholarly communication to determine what knowledge is valued, recognized, and utilized. By empirically examining whether knowledge production in Med Ed research draws from multiple disciplines and research areas, or whether it primarily draws on the knowledge generated internally within the field of Med Ed, this article explores whether the characterisation of Med Ed research as interdisciplinary is substantiated. A citation analysis of 1412 references from research articles published in 2017 in the top five Med Ed journals was undertaken. A typology of six knowledge clusters was inductively developed. Findings show that the field of Med Ed research draws predominantly from two knowledge clusters: the Applied Health Research cluster (made of clinical and health services research), which represents 41% of the references, and the Med Ed research cluster, which represents 40% of the references. These two clusters cover 81% of all references in our sample, leaving 19% distributed among the other knowledge clusters (i.e., Education, disciplinary, interdisciplinary and topic centered research). The quasi-hegemonic position held by the Applied Health and Med Ed research clusters confines the other sources of knowledge to a peripheral role within the Med Ed research field. Our findings suggest that the assumption that Med Ed research is an interdisciplinary field is not convincingly supported by empirical data and that the knowledge entering Med Ed comes mostly from the health research domain.

## Introduction

Is the field of medical education (Med Ed) research an interdisciplinary field? This question may sound odd to members of the field as it is generally presumed that Med Ed research draws on the insights, methods, and knowledge from multiple disciplines and research domains (e.g. Sociology, Anthropology, Education, Humanities, Psychology) (Albert et al. 2020). This common view of Med Ed research is echoed and reinforced by the narrative used by leading Med Ed departments and research centres to describe their activities. Words and expressions such as “interdisciplinarity,” “multidisciplinary perspective,” and “opportunities for interdisciplinary collaboration” are frequently used to depict their mission, goals, and the academic training they provide to their students.^1^ Researchers in Med Ed also often characterise the field as being a hybrid domain, building on various methodologies and disciplines (Gruppen 2014; Gwee et al. 2013; O’Sullivan et al. 2010; Teodorczuk et al. 2017).

In this article, we examine the assumption that Med Ed research is an interdisciplinary field. This wide-spread assumption has not yet been investigated. To date, there is no empirical evidence that either supports or calls into question that the field is interdisciplinary. Answering this question may impact the development of Med Ed research as members of the field will be able to make an informed decision about how they would like to see the field develop. In this article, we define interdisciplinarity, following the National Academy of Science’s definition (2005), as communication and collaboration between researchers across academic disciplines and research domains.

One effective method to investigate whether Med Ed research is interdisciplinary is to conduct a bibliometric analysis (Larivière and Gingras 2014) of articles and journals cited by Med Ed researchers. Bibliometrics offer a “set of methods and measures for studying the structure and process of scholarly communication” (Borgman and Furner 2002, p. 2). Bibliometric data can shed light on the knowledge that informs Med Ed academics in their published work and, concurrently, trace the contour of the intellectual landscape of the Med Ed research field. By using bibliometric data, we are studying cross-disciplinary communication. We are not attempting to make any claims about the collaborative dimension of interdisciplinarity, as that is beyond the scope of this methodology and this paper. We examined one facet of cross-disciplinary communication, which is the flow of ideas, concepts, and knowledge from other disciplines into the Med Ed field.

A growing body of research shows that cross-disciplinary exchange is a common feature of academic life. Rob Moore coined the term “routine interdisciplinary” to capture this practice (2009). Jacobs and Frickel (2009), Jacobs (2014), Larivieres and Gingras (2014), and Van Noorden (2015) used bibliometrics to show that it is a customary practice for scientists to cite the work from colleagues outside their discipline. We build on this body of work in this study by examining Med Ed researchers’ distinct citation practice. We use these citation practices as a way to trace which disciplines and domains of knowledge are drawn upon by Med Ed researchers to inform their work.

## Method

### Journals and research articles sampling rationale and procedure

The first step in our bibliometric analysis was identifying the five Med Ed journals with the highest impact factor by using the 2017 Journal Citation Reports (JCR) (2018 was not available at the time of data collection). The JCR category used was “Education, Scientific Disciplines,” which is where Med Ed journals are classified by Clarivate Analytics. The five journals with the highest impact factor in Med Ed (at the time we conducted our research) were: *Academic Medicine*, *Medical Education*, *Advances in Health Sciences Education*, *Medical Teacher*, and *BMC Medical Education* (see Table 1). Our goal was not to create a sample of journals representative of the whole range of publications in the Med Ed research field, but to select the most cited journals, i.e., those that are the most influential in the field.

**Table 1:** The five most cited journals in medical education research in 2017. *Source*: Journal Citation Reports (JCR) Year: 2017 Selected Editions: SCIE Selected Categories: ‘EDUCATION,

Journals	Total research articles published in 2017	10% of research articles published in 2017
Academic Medicine (JIF*: 4.8)	134	13
Medical Education (JIF: 4.4)	81	8
Advances in Health Sciences Education (JIF: 2.5)	66	7
Medical Teacher (JIF: 2.4)	124	12
BMC Medical Education (JIF: 1.5)	242	24
Total	647	64

SCIENTIFIC DISCIPLINES’ Selected Category Scheme: WoS

*JIF  journal impact factor

The second step was selecting a sample of articles published within these five journals. Since the goal of our project was to study the patterns of knowledge circulation within Med Ed research, we included *research* articles published in 2017 in the five journals. The research articles from 2017 from each journal were exported from Web of Science and cross-checked manually with each journal’s Table of Contents. For feasibility, we included only a subset of the total research articles published in 2017. Using a random number generator (random.org), we selected 10% of the research articles published in each journal (see Table 1). The procedure we followed for each journal is exemplified by the steps we took to select articles from the journal *Academic Medicine* which published 134 research articles in 2017. First, we numbered 1–134 all the research articles published in the journal in 2017. Second, we set the number generator minimum as 1 and maximum as 134, and then we generated 13 random numbers (i.e., 10% of the 134 research articles). For our study dataset, we used the 13 articles that matched the randomly generated numbers. We repeated this procedure for all selected journals. In total, 64 articles were selected to be included in our sample across the five journals. Table 1 outlines the number of research articles published in each of the five targeted journals in 2017 and the number of articles randomly selected per journal based on the 10% ratio. Reviews, commentaries, letters, editorials, and other non-primary research formats were excluded from our sample as they are not research (e.g. *Academic Medicine* Last Page, *Medical Teacher* Twelve Tips, *Advances in Health Sciences Education* Reflection articles, *Medical Education* When I Say).

We decided to sample 10% of the research articles published in each journal for feasibility reasons. Our goal was not to be exhaustive but to ensure a reasonable representation of the work without skewing the selection in favour of any one research area or discipline. We have no reason to believe that the articles included in our sample are meaningfully different in terms of reference patterns from those not included.

### References sampling

The third methodological step was constructing our references dataset from the 64 randomly selected research articles. We exported the references for each of the 64 articles from Web of Science and cross-checked manually with the article PDFs to ensure all references were captured. We included references (n = 1412) from peer-reviewed journals as our unit of analysis.

**Fig. 1:**
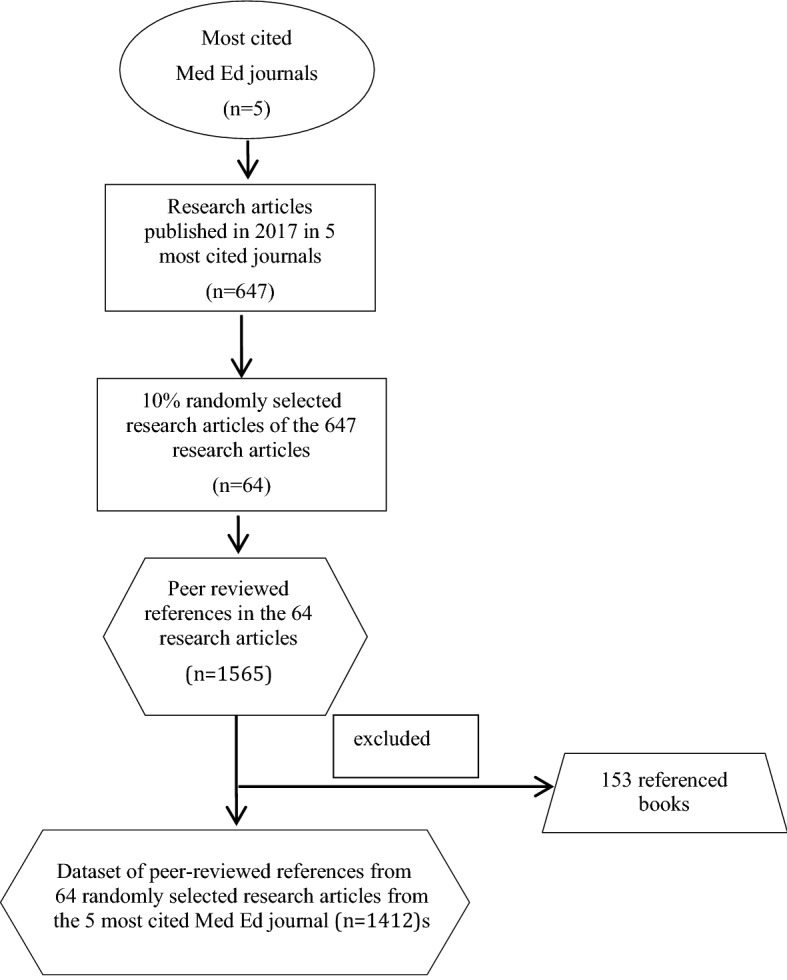
Procedure used to construct the dataset of peer-reviewed cited references from the five most cited Med Ed journals

In total, 153 references cited books across the 64 articles included in our sample, in comparison with 1412 references which cited peer-reviewed journal articles. The ratio of book references to journal references is therefore: 1412:153 which is just over nine. Proportionally, this means that there are nearly nine times more references from journals than from books. We did, however, review the titles of these books as part of our initial analysis. There was no particular trend arising from the book references that significantly alter the patterns observed across the articles. For the remainder of this paper, we will focus on the analysis of the 1412 journal articles referenced, which was our dataset. Figure 1 outlines the steps we took constructing our references dataset.

### Data analysis

To analyse the references and examine which disciplines Med Ed researchers draw from, we inductively developed a typology of six knowledge orientations, which we labelled knowledge clusters (see Table 2). These knowledge clusters were developed by examining the “Aims and Scope” of the journals cited by the 1412 references. All journals have a web page dedicated to their “Aims and Scope” where they describe their mandate and the type(s) of research they consider in alignment with their editorial orientation. The details provided typically list what discipline(s), topic(s), research area(s), and method(s) fall within their scope. The categorisation procedure we followed is similar to the one followed by qualitative researchers when conducting thematic analysis: in both types of research, categories, or clusters, are gradually developed through an iterative process of inclusion, exclusion, expansion, and division. Clusters take their final shape only when the analysis of new data (quotes in qualitative research, journals in our study) is completed. This inductive procedure should not be confused with the statistical cluster analysis method used in quantitative studies.

**Table 2:** Six inductively developed knowledge clusters

Knowledge clusters	Description of knowledge clusters and name of the 6 most cited journals by Med Ed researchers in each cluster
1. Disciplinary and institutionalised research fields	Includes disciplinary journals (e.g. Psychology, Biology, Sociology) and journals focusing on well-established research areas (e.g. Business and Management, Organization Studies, Cognitive Sciences) 6 most cited journals: *Journal of Personality and Social Psychology*; *Qualitative Research in Psychology*; *Educational Psychologist*; *American Psychologist*; *Journal of Clinical Psychology*; *Psychological Reports*
2. Topic centered (non health)	Includes journals focusing on a specific topic, but not health related (e.g. accident prevention, industrial ergonomic, migration and human security) 6 most cited journals: *Educational and Psychological Measurement*; *Assessment*; *Journal of Vocational Behavior*; *Journal of Research in Personality*; *Milbank Quarterly*; *Personality and Individual Differences*
3. Education	Includes journals focusing on education research (includes higher education and profession/science education) 6 most cited journals: *American Journal of Physiology—Advances in Physiology Education*; *Review of Educational Research*; *Planning for Higher Education*; *CBE-Life Sciences Education*; *Studies in Continuing Education*; *Learning and Instruction*
4. Medical education	Includes journals focusing on any aspect of medical and health professions education 6 most cited journals: *Academic Medicine*; *Medical Education*; *Medical Teacher*; *BMC Medical Education*; *Advances in Health Sciences Education*; *Journal of Graduate Medical Education*
5. Interdisciplinary health	Includes interdisciplinary journals focusing on health-related issues 6 most cited journals: *PLoS ONE; Social Science and Medicine*; *Simulation in Healthcare*; *Health Affairs*; *Journal of Religion and Health*; *Journal of Women’s Health*
6. Applied health research (mainly Health Services Research [HSR] and clinical research)	Includes journals focusing on applied health research, mainly Health Services Research journals (HSR) and clinical journals 6 most cited journals: *Journal of the American Medical Association (JAMA)*; *Journal of General Internal Medicine*; *New England Journal of Medicine (NEJM)*; *British Medical Journal* (*BMJ)*; *Medical Journal of Australia*; *Annals of Internal Medicine*

The six inductively developed knowledge clusters served as our conceptual map to categorise the 1412 references. For example, a reference from the journals *Journal of the American Medical Association (JAMA)* or the *New England Journal of Medicine* (*NEJM*) was classified within the Applied Health Research knowledge cluster because *JAMA* and *NEJM*, based on their aims and scope, are two journals whose primary research orientation is applied health research (in contrast, for example, to basic or disciplinary health research). Another example is the journal *Simulation in Healthcare*. We categorised this journal within the Interdisciplinary Health knowledge cluster because its main focus is healthcare simulation technology (which is a research topic, not a discipline) and it defines itself as a multidisciplinary publication. In cases where the information posted on the aims and scope web page was insufficiently detailed or unclear, members of the research team (MA and SL) read articles published in the recent issues of the referenced journals before categorising. In order to provide as much detail as possible on the journal categorisation, we list in Table 2 the six journals with the highest number of references for each cluster. The detailed list will help elucidate the groupings made therein and the knowledge orientation of each cluster.

We chose to design our groupings of journals as inductively developed “clusters” instead of “categories” since the type of data we are working with (i.e., references, journals, and ultimately areas of knowledge production) is inherently porous. The notion of cluster, more than category, better reflects this porosity. All journals within a cluster share a key characteristic (for example, being topic-centered, disciplinary, or education-focused); however, a number of journals may also share aspects with, or overlap onto, a neighboring cluster. For example, the journal *Educational and Psychological Measurement* was included in the Topic-Centered knowledge cluster because its primary focus is measurement; however, articles in this journal may tangentially address educational or psychological issues. The assignment of any journal to a knowledge cluster was based on its predominant characteristics. The clustering was performed by MA and SL.

### Findings

#### Citation patterns in medical education journals

**Fig. 2:**
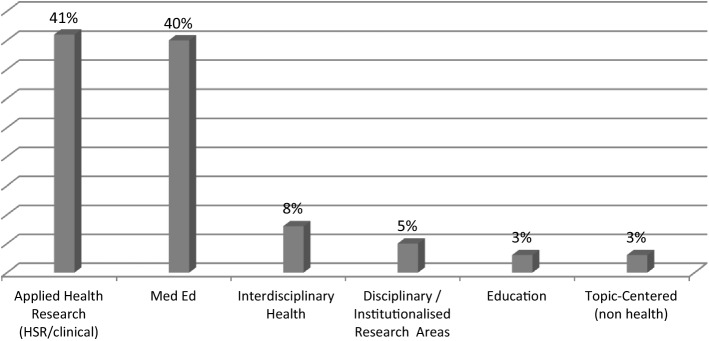
Distribution of peer-reviewed references (n = 1412) per knowledge cluster. Data presented in %

The most striking finding is the steep discrepancy between, on the one hand, the Applied Health Research and Med Ed knowledge clusters and, on the other hand, the other four clusters (see Fig. 2). The Applied Health and Med Ed clusters cover 81% of all references, leaving 19% distributed among the other knowledge clusters. This pattern suggests that the field of Med Ed research stands predominantly on two knowledge pillars. These pillars are health services research and clinical research (which represent 41% of the references) and Med Ed (which represents 40% of the references). The quasi-hegemonic position held by these two knowledge clusters confines the other sources of knowledge to a peripheral role within the Med Ed research field.

When Med Ed researchers seek knowledge from outside their field and the applied health research field, they primarily turn to interdisciplinary health research (see Interdisciplinary Health in Fig. 2). The journals included in this cluster cover a wide spectrum of topics, from drugs and alcohol dependence to women’s health, health policy, simulation, and religion and health. One key characteristic of the journals in this cluster is that some of the articles draw on the knowledge developed in the core disciplines and institutionalised research areas (organisation studies, cognitive sciences, sociology, etc.). While their mandate is usually not to expand theory (but rather to report on empirical research), it is not uncommon for a number of these journals, such as *Social Science & Medicine*, to publish articles offering a conceptual understanding of phenomena. Therefore, through the journals included in the Interdisciplinary Health knowledge cluster, Med Ed researchers have a connection with a more comprehensive source of knowledge that is both basic and applied.

As shown in Fig. 2, the Interdisciplinary Health knowledge cluster stands in third position in terms of its overall volume of references. Yet, this volume represents only 8% (n = 115 references) of the 1412 references in our sample used by Med Ed researchers. It follows that the capacity of this body of knowledge to influence the Med Ed academic culture is likely to be relatively marginal.

The bodies of literature cited by Med Ed researchers decreases substantially when these references come from journals outside health research. The next three clusters in Fig. 2 (the Disciplinary and Institutionalised Research Areas cluster, the Education cluster, and the Topic-Centered (non-health) cluster) respectively represent only 5%, 3%, and 3% of all references. One would expect that Med Ed researchers draw upon these research areas more—especially education, as it is a cognate field. Our data suggest that, to the contrary, Med Ed researchers tend to marginally engage with this literature.

In order to paint a more detailed picture of the disciplinary knowledge entering the Med Ed intellectual space, we examine the Disciplinary and Institutionalised Research Areas knowledge cluster more closely. We noticed that one discipline (psychology) largely exceeded the others, which has the effect of further narrowing down the range of disciplinary knowledge entering the Med Ed field. Among the 77 references contained in this cluster, 55 (71%) are from psychology journals (including seven from psychology journals applied to education). The remaining references are distributed across nine disciplines and research areas: sociology (n = 6), business, management and organisations studies (n = 5), social psychology (n = 3), biology (n = 2), the humanities (n = 2), neuropsychology (n = 1), physiology (n = 1), statistics (n = 1), and cognitive science (n = 1).

One of the common goals of the journals included in the Disciplinary and Institutionalised Research Areas knowledge cluster is to advance both basic and applied disciplinary and interdisciplinary knowledge through cross-communication between empirical research and theory. Based on the citation patterns revealed by our data, it seems that this mix of disciplinary, basic/applied, and theoretical knowledge does not easily find its way into the Med Ed research field. While psychology may be utilized by Med Ed researchers, the other disciplines are essentially absent.

## Discussion

Our findings suggest that when Med Ed researchers seek knowledge from outside their field, they predominantly draw from one research area (i.e., applied health). This practice seems to show some discrepancy in regard to the commonly accepted definition of interdisciplinarity, which emphasizes knowledge flow between various research communities (National Academy of Science 2005). The high percentage of citations from just one knowledge cluster outside Med Ed suggests that the knowledge landscape of the field could be portrayed as being only tangentially interdisciplinary. With 41% of the references coming from health services research and clinical journals, the academic group with whom Med Ed researchers have the closest connection is the applied health research community. This privileged connection risks excluding other research communities and these other robust bodies of knowledge. These other research communities are the social scientists, the natural scientists, and the humanities scholars. It seems, therefore, that interdisciplinarity for Med Ed researchers means, first and foremost, drawing on and being inspired by clinical and health services research and by the medical epistemic culture underpinning these knowledge domains. Basic, (inter)-disciplinary, and theory-based knowledge seems to be infrequently used by Med Ed researchers and therefore is unlikely to influence the field in a meaningful way. In light of this, one wonders how the Med Ed research field may best be characterised: as an interdisciplinary sub-field of education or as a sub-field of health research? The difference is potentially subtle, but reflecting on this positioning draws attention to how we might think about the dynamics of knowledge production and the future of Med Ed.

If Med Ed researchers predominantly use knowledge developed in health services research and the clinical sciences, it is legitimate to ask whether there could be a misalignment between the body of knowledge drawn from and their research object—education—which is primarily a social science object. If education is a multifaceted phenomenon, how can it be comprehensively studied if researchers draw on a relatively narrow range of knowledge sources, methods, and approaches? Specifically, how can the sociological, psychological, political, cultural, and historical dimensions embedded in the practice of education be studied and understood, without substantive inputs from the academic disciplines focusing on understanding these aspects (Bridges 2017; Furlong 2013)?

Further, the lack of engagement with wider developments in disciplinary knowledge may raise a number of challenges: Med Ed researchers may not have an in-depth understanding of the literature coming from these fields and have difficulty teaching disciplinary knowledge to their students, further hindering knowledge communication with scholars outside Med Ed. Because of the health orientation and relative insularity of the field (as our data suggest), Med Ed researchers could also find themselves asking questions that researchers and practitioners within the Med Ed field may find interesting and novel, but that might have already been investigated by researchers in the social and natural sciences. This would be a lost opportunity to build Med Ed knowledge upon existing foundations, and could potentially undermine the status of the Med Ed field in the broader academic market where it is common practice to borrow ideas, concepts, and methods from other disciplines (Albert et al. 2020; Jacobs 2014, Larivière and Gingras 2014; Moore 2011). Research fields with a low participation in knowledge exchange across the university may potentially be at risk of being left behind when new theoretical and methodological developments occur (Jacobs 2014). Being “out of the loop” may also result in Med Ed researchers potentially applying a theoretical framing or methodological approach that has already been debated, dismissed, or evolved in other disciplines.

## Conclusion

The goal of this study was to examine the widespread assumption that Med Ed research is an interdisciplinary field. Our findings show that this belief is not convincingly supported by empirical data and that the knowledge entering, circulating, and informing Med Ed research comes mostly from the health research domain. It behooves members of the Med Ed field to reflect on the current knowledge flow: is the health orientation of the field appropriate and sufficient to pursue education research in health care or is a diversification of knowledge needed? We see this as a contribution to the understanding of what the Med Ed field is, and what could be done to further its development.

Our study is not without limitations. A larger sample may have offered a more refined picture of the citation practices of Med Ed researchers. Also, since we have not used a comparative design, we cannot interpret our findings in comparison with the knowledge flow occurring in other research communities. This is something worth studying in future projects.

Further research should also explore the reasons why Med Ed researchers tend to predominantly cite literature from health rather than from non-health disciplines. Perhaps Med Ed researchers build their rationale for education research from the observation of clinical problems (i.e. the needs of a particular patient population indicate a need for further clinical education), but do not go far beyond the clinical rationale in their literature searches. Another reason could be that clinical and health services research journals are the most familiar to Med Ed researchers, perhaps the most easily accessible and discussed in their academic environment. It could also be that researchers in the Med Ed field view Education and the social sciences as having little to contribute to education in health care settings. Investigating these questions will help develop a better understanding of the factors influencing knowledge production in Med Ed research and situate the field among other education research fields. The types of knowledge drawn upon have implications for how education interventions are designed and how we train future generations of Med Ed researchers.

## Footnotes

1 The two following quotes illustrate the interdisciplinary narrative used by Med Ed departments and research centres to describe the nature of their core activities: “The Centre for Education Research and Innovation is an interdisciplinary, collaborative research group exploring health professions education research” (Centre for Education Research and Innovation, Western University 2019). “Health sciences education research draws on theories and methodologies from across myriad research traditions.” (Centre for Medical Education, McGill University 2019). Other examples of interdisciplinary narratives can be found on the web site of the School of Health Profession Education, Maastricht University (2019), the Centre for Health Education Scholarship, University of British Columbia (2019), the Health Professions Education Research PhD Program, University of Toronto (2019), the Department of Innovation in Medical Education, University of Ottawa (2019) and Chang Gung Medical Education Research Center, Chang Gung University (2019).
